# Diffusion of the Digital Health Self-Tracking Movement in Canada: Results of a National Survey

**DOI:** 10.2196/jmir.9388

**Published:** 2018-05-02

**Authors:** Guy Paré, Chad Leaver, Claire Bourget

**Affiliations:** ^1^ Research Chair in Digital Health HEC Montreal Montreal, QC Canada; ^2^ Canada Health Infoway Toronto, ON Canada; ^3^ CEFRIO Montreal, QC Canada

**Keywords:** self-tracking, quantified-self, wearable devices, activity trackers, survey methodology

## Abstract

**Background:**

With the ever-increasing availability of mobile apps, consumer wearables, and smart medical devices, more and more individuals are self-tracking and managing their personal health data.

**Objective:**

The aim of this study was to investigate the diffusion of the digital self-tracking movement in Canada. It provides a comprehensive, yet detailed account of this phenomenon. It examines the profile of digital self-trackers, traditional self-trackers, and nontrackers, further investigating the primary motivations for self-tracking and reasons for nontracking; barriers to adoption of connected care technologies; users’ appreciation of their self-tracking devices, including what they perceive to be the main benefits; factors that influence people’s intention to continue using connected care technologies in the future; and the reasons for usage discontinuance.

**Methods:**

We conducted an online survey with a sample of 4109 Canadian adults, one of the largest ever. To ensure a representative sample, quota method was used (gender, age), following stratification by region. The maximum margin of error is estimated at 1.6%, 19 times out of 20.

**Results:**

Our findings reveal that 66.20% (2720/4109) of our respondents regularly self-track one or more aspects of their health. About one in 4 respondents (1014/4109, 24.68%) currently owns a wearable or smart medical device, and 57.20% (580/1014) use their devices on a regular basis for self-tracking purposes. Digital self-trackers are typically young or mature adults, healthy, employed, university educated, with an annual family income of over $80,000 CAD. The most popular reported device is the fitness tracker or smartwatch that can capture a range of parameters. Currently, mobile apps and digital self-tracking devices are mainly used to monitor physical activity (856/1669, 51.13%), nutrition (545/1669, 32.65%), sleep patterns (482/1669, 28.88%) and, to a much lesser extent, cardiovascular and pulmonary biomarkers (215/1669, 12.88%), medication intake (126/1669, 7.55%), and glucose level (79/1669, 4.73%). Most users of connected care technologies (481/580, 83.0%) are highly satisfied and 88.2% (511/580) intend to continue using their apps and devices in the future. A majority said smart digital devices have allowed them to maintain or improve their health condition (398/580, 68.5%) and to be better informed about their health in general (387/580, 66.6%). About 33.80% of our sample (1389/4109) is composed of people who do not monitor their health or well-being on a regular basis.

**Conclusions:**

Our study shows an opportunity to advance the health of Canadians through connected care technologies. Our findings can be used to set baseline information for future research on the rise of digital health self-tracking and its impacts. Although the use of mobile apps, consumer wearables, and smart medical devices could potentially benefit the growing population of patients with chronic conditions, the question remains as to whether it will diffuse broadly beyond early adopters and across cost inequities.

## Introduction

In *The patient will see you now*, Eric Topol describes mobile phones as the Gutenberg of health care [1]. He argues that small mobile devices with wireless connectivity will prove to be the transformative catalyst for advancing toward the future of medicine. Although the mobile phone remains the device of choice for most individuals, technology manufacturers are creating a future of consumer wearables and smart medical devices that promise to help people live healthier lives [2]. Several such devices are currently available, including physical activity trackers that measure fitness-related metrics such as distance walked or ran; smart forks that vibrate when people eat too fast; smart toothbrushes with 3D motion sensors that monitor brushing performance; and smart clothes (eg, biometric shirts) that measure a person’s breathing, pulse, calories, and sleep patterns. Recent estimates predict that 5.2 billion consumer smart devices are in use globally in 2017, setting the stage for an estimated 12.9 billion devices to be deployed by 2020 [3]. Other forecasts indicate that the consumer wearable device market value will reach US $41 billion by 2020 from US $2 billion in 2014 [4].

The need to attach personal numeric data to day-to-day activities such as eating, sleeping, and exercising is called the “quantified-self” movement [5]. Proponents of this movement believe that if they can measure an aspect of their life on a regular basis, they can find a way to improve it [6]. Computers can facilitate self-tracking because of advances in sensor technologies, ubiquity of access to information brought by the Internet, and improvements in user-friendly systems and interfaces [7]. Prior research shows that the measurement of one’s daily activities with the assistance of mobile devices provides an advantage with respect to automatic and aggregated data compilation. People have limited memory and capacity to accurately and consistently track computational data about their behaviors such as counting the number of steps throughout the day [7-9]. Another advantage is that data from wearable sensors and smart medical devices can generate automated analytics over time, aggregating personally relevant feedback, which may, in turn, contribute to the sustainable use of digital devices [10,11].

Many experts say the rise of the Internet of Things will bring the next revolution in digital health [12]. Recent surveys on the adoption and impact of consumer digital health technologies reveal important insight about the current state and the trajectory of the purported potential. For instance, a survey of 2225 US adults reveals that the use of wearable self-tracking devices has doubled from 2014 (9%) to 2016 (21%) [13]. This study also indicates that millennials (aged 18-34 years) are the most prevalent users (36%). Another US-based survey (n=2025) on telemedicine, wearables, and postdischarge care found that 27% of adults own a self-tracking device and that 78% would want their doctor to have access to data from their wearables [14]. In Europe, a national survey of 1005 French citizens (aged 15+ years) reveals that 11% possess a health wearable or smart medical device, and 30% of nonusers have a firm intention to buy one in the next 12 months [15]. Finally, a 2016 online survey of more than 20,000 consumers (aged 15+ years) from 16 countries reported that 33% of respondents tracked their physical activity via a mobile app and a fitness band, clip, or smartwatch [16].

Although the abovementioned surveys set relevant baseline information, they do not provide a comprehensive and detailed account of the digital self-tracking movement. Specifically, no prior empirical research has attempted to investigate the sociodemographic and preference profile of digital self-trackers, traditional self-trackers, and nontrackers; the primary reasons for self-tracking and nontracking; the barriers to adoption of smart and connected health devices; users’ perceived benefits of these devices; the factors that influence people’s intention to continue using connected care technologies in the future; and the reasons for usage discontinuance that remain largely unknown. This study aims to fill this important gap and presents a timely and relevant integration of these issues, which may inform technology manufacturers, health care providers (HCPs), and policy makers’ perceptions and future decisions in this area.

## Methods

### Study Design and Sample

In this section, we report the online survey that was conducted in according with the Checklist for Reporting Results of Internet E-Surveys checklist [17]. We first developed a comprehensive questionnaire instrument to administer with the general Canadian population in 2017. The instrument was based on a review of the extant literature on mobile health (mHealth) and digital self-tracking and was originally designed in French and back translated to English. The questionnaire was pretested during face-to-face interviews with 16 adults representative of the Canadian population in terms of age, gender, and language. Some minor adjustments were made to the questionnaire following this initial step.

The online survey was administered by AC Nielsen Company of Canada. The sample used for this research was the company’s proprietary online panel, known as the Harris Panel. This panel is one of the largest, most representative, and best profiled panels in Canada. To begin survey administration, panel members were invited to participate in the study by email. Once participants clicked on the URL provided in the email letter, they were screened for the following eligibility criteria: (1) Canadian resident, (2) aged 18 years or older, and (3) spoke English or French. Those who were eligible read an informed consent form that emphasized the anonymity and confidentiality of respondents and advised that by completing the questionnaire, they are providing their consent to participate. All study procedures were approved by the HEC Montreal’s research ethics committee. To ensure a representative sample, the quota method was used (gender, age), following stratification by Canadian geographic regions.

Survey respondents were able to enter the survey at any point during the data collection period, that is, from January 11, 2017 to February 2, 2017. Respondents who partially completed the survey were able to exit the questionnaire and return at a later time to enter additional data. This could be done as many times as necessary. In accessing the online survey, respondents were assigned a unique identifier and passcode that allowed them access their data until the survey was finished. Participants were rewarded points for survey completion. Rewards for completing AC Nielsen surveys range in value from $5 CAD to $75 CAD. Standard options include gift cards and merchandise (eg, Amazon, iTunes, magazine subscriptions, Starbucks, Wal-Mart, and a variety of restaurant gift cards).

### Survey Items

Gender, age, region, gross family income, education, occupation, and use of mobile phones and digital tablets were assessed by standard survey items administered in other international surveys [13-16]. Overall health status was obtained by asking participants to self-rate their own health on a scale from 1=poor or fair to 5=very good or excellent. This single-item measure has been used extensively worldwide and represents a valid and acceptable measure [18]. We also asked participants if they had one or several of the following chronic conditions: (1) diabetes, (2) high blood pressure, (3) obesity, (4) cardiovascular disease, (5) lung or respiratory airway disease, (6) cancer, (7) bone or muscular disease, (8) disease of nervous system, (9) mental disorder, (10) chronic infectious disease, and (11) addiction to tobacco or drugs.

Familiarity with connected care technologies was measured by asking “How familiar are you with consumer health wearables and smart medical devices?” using a 5-point Likert scale, where 1=not much at all and 5=extremely. We then asked, “Which of the following devices do you own?” using descriptive nonbrand terms for 13 specific devices commonly listed in the extant literature and available in Canada (see Results section). For each device they own, respondents were then asked how often they use it using a 7-point scale, where 1=once a month or less and 7=many times each day.

Motivations for using digital health self-tracking devices were measured with 10 items developed for this study using 5-point Likert scales, where 1=not at all and 5=very strongly. Items were derived from prior surveys on consumer digital health [12-16]. Examples of items include “know myself better,” “give me daily encouragement toward reaching my personal goals,” “better follow the treatment plan prescribed by my physician,” and “break a bad habit related to my health.” Data-sharing behaviors were assessed with a single item asking “Do you ever share with other people the data stored in your device or mobile app?” When answering “yes,” respondents were then asked with whom (eg, family members, friends, family doctor, pharmacist, or personal trainer).

Respondents’ appreciation of wearables and smart devices were captured with five variables. Measures for perceived usefulness (7 items) and ease of use (4 items) were adapted to the context of this study from Davis [19]. For their part, user satisfaction (3 items), confirmation of initial expectations (3 items), and intention to continue using wearables and smart devices (3 items) were adapted from Bhattacherjee [20] and Hong et al [21]. All five variables were assessed using 5-point Likert scales, where 1=strongly disagree and 5=strongly agree.

Finally, we asked respondents (when applicable) why they did not currently possess health wearables or smart devices. We developed a list of 10 reasons (see Results section), and respondents only checked those that applied to their personal situation. In a similar fashion, we developed a list of 11 items (see next section) that correspond to the reasons why consumers stopped using their wearables and smart devices at some point. Both lists of items were derived from prior surveys on consumer digital health [12-16]. The complete online survey instrument is provided in Multimedia Appendix 1.

### Data Analyses

In line with our research objectives, we analyzed the entire sample as well as specific subgroups. General trends regarding ownership and use of connected care technologies are analyzed with descriptive statistics (mean, SD, percentage), comparisons between self-trackers and nontrackers are analyzed with multinomial logistic regression tests, and users’ appreciation of digital self-tracking devices is analyzed using Pearson correlation tests and partial least squares (PLS) multiple regression analyses. Analyses are performed using the SPSS (IBM Corp) version 23 software and the SmartPLS (SmartPLS GmbH) version 3.2.7 software.

## Results

### Profile of the Sample

Our sample is composed of 4109 adults. The maximum margin of error is estimated at 1.6%, 19 times out of 20. Table 1 presents the profile of the sample according to usual sociodemographic variables, in comparison with the total Canadian population. The sample was composed of 2118 men, representing 51.55%. In terms of age, 27.84% of all respondents (1144/4109) were millennials (18-34 years), whereas 35.17% (1445/4109) consisted of baby boomers (55+ years). As expected, the majority of respondents were from the two largest Canadian provinces, namely, Ontario (1575/4109, 38.33%) and Quebec (986/4109, 24.00%). About 1 out of 5 respondents had a gross family annual income of less than $40,000 CAD, whereas 35.58% (1462/4109) had annual family incomes superior to $80,000 CAD. Our survey participants were more educated than the Canadian population according to data from the 2016 national census. Almost half of the respondents had a university degree compared with 28.70% for the whole population, 6 out of 10 respondents were workers (2386/4109), less than 4% (3.68%, 151/4109) were students, and slightly over 23% (22.8%, 937/4109) were retired. Overall, our data indicate that, except for education, the sociodemographic profile of our respondents is representative of the adult population in Canada.

In terms of health status, less than 10% of all respondents (9.78%, 402/4109) perceived themselves to be in poor or fair condition, whereas 50.38% (2070/4109) said they were in good health, and 39.84% (1637/4109) perceived themselves in very good or excellent health.

**Table 1:** Profile of the sample and comparisons with the Canadian population.

Characteristics		Sample (N=4109), n (%)	Canadian population (N=35,151,730), n (%)
**Gender**			
	Male	2118 (51.55)	17,264,200 (49.11)^a^
	Female	1991 (49.45)	17,887,530 (50.89)^a^
**Age (years)**			
	18-34	1144 (27.84)	6,858,075 (25.27)^a^
	35-54	1520 (36.99)	9,581,540 (27.28)^a^
	55+	1445 (35.17)	10,846,380 (30.86)^a^
**Region**			
	Atlantic provinces	293 (7.13)	2,385,779 (6.58)^a^
	Quebec	986 (24.00)	8,321,888 (22.95)^a^
	Ontario	1575 (38.33)	13,976,320 (38.54)^a^
	Manitoba and Saskatchewan	266 (6.47)	2,466,703 (6.80)^a^
	Alberta	437 (10.64)	4,236,376 (11.68)^a^
	British Columbia and Northwest Territories	552 (13.43)	4,802,275 (13.24)^a^
**Gross family income^b^ ($ CAD)**			
	<$20k	268 (6.52)	8,558,000 (29.88)^a^
	≥$20k and <$40k	583 (14.19)	7,014,015 (24.48)^a^
	≥$40k and <$60k	614 (14.94)	5,006,820 (17.48)^a^
	≥$60k and <$80k	561 (13.65)	2,926,920 (10.22)^a^
	≥$80k and <$100k	498 (12.12)	1,716,175 (5.99)^a^
	≥$100k	964 (23.46)	2,266,600 (7.91)^a^
**Education level**			
	High school or college	2051 (51.13)	18,730,750 (65.39)^a^
	Undergraduate	1300 (32.41)	6,659,615 (23.25)^a^
	Graduate	660 (16.45)	1,562,555 (5.45)^a^
**Occupation**			
	Workers	2386 (58.86)	17,230,040 (60.15)^a^
	Students	151 (3.72)	19,992,283 (6.99)^a^
	Retirees	937 (23.11)	4,912,278 (17.15)^a^
	Other	580 (14.31)	4,284,996 (15.96)^a^
**Perceived health status**			
	Bad or average	402 (9.78)	3,443,000 (12.00)^c^
	Good	2070 (50.38)	9,561,713 (29.00)
	Very good or excellent	1637 (39.84)	18,714,100 (59.00)^c^
**Chronic diseases**			
	Yes	1281 (31.89)	12,053,150 (38.00)^c^
	No	2735 (68.11)	19,665,665 (62.00)^c^
**Language used to complete the questionnaire**			
	English	3644 (88.68)	—
	French	465 (11.32)	—

^a^Statistics Canada Census 2016.

^b^The median total income in Canada was $80,940 CAD in 2015 according to the Statistics Canada Census 2016.

^c^Health Canada Survey 2014.

Aligned to general population estimates for Canada, the majority of respondents (2735/4109, 68.11%) reported no chronic conditions. The most common self-reported chronic conditions were hypertension, diabetes, and obesity.

### Use of Mobile Devices

Our findings indicate that 78.10% of all respondents (3209/4109) owned a mobile phone (eg, Apple iPhone, Samsung Galaxy, Google Nexus, Microsoft Lumia, or Sony Xperia) and used it to download mobile apps, among other things. Our results also show that 56.88% of our respondents (2337/4109) owned a digital tablet (eg, Apple iPad, Samsung Galaxy Tab, Google Nexus Tablet, or Sony Xperia Tablet). These statistics are similar to those reported by the Canadian Radio-television and Telecommunications Commission, which found that in 2016, 73% of Canadian adults owned a mobile phone, and 52% owned a tablet [22]. Overall, 86.01% of our respondents (3534/4109) used either a mobile phone or a tablet, whereas 48.97% (2012/4109) reported owning both devices. As expected, age is negatively associated with use of a mobile device (χ^2^_4_=389.3; *P*<.001); 56.24% of millennials (18-34 years) using both devices compared with 37.11% among baby boomers (55+ years).

### Self-Tracking Behaviors

We defined three self-tracking profiles for the study. Respondents that regularly track one or more aspect of their health or well-being using connected care technologies, that is, mHealth apps, consumer wearables (eg, fitness trackers), and smart medical devices (eg, blood pressure monitors) were defined as “digital self-trackers.” Respondents who regularly monitor one or more aspect of their health using manual tools (other than a mobile app or smart device) such as simply recording the information in writing (on paper, or in a journal or notebook) or by remembering the information were defined as “traditional self-trackers.” The remaining respondents reporting that they do not regularly monitor any aspect of their personal health or well-being were defined as “nontrackers.”

Table 2 reveals that whether through digital or traditional means, the prevalence of self-trackers in Canada is about two-thirds (2720/4109, 66.20%), a number that is similar to recent estimates from the United States [23]. Our sample is composed of two distinct groups of self-trackers. The first, digital self-trackers comprise 40.61% (1669/4109) of our sample and 61.36% of all self-trackers (1669/2720). This group comprises as many men as women who are, for the most part, active members of the workforce. A majority of digital self-trackers are young or mature adults (18-34 years), highly-educated, and wealthy individuals (average gross family income $80,000 CAD), and people who perceive themselves to be in good or very good health. More than 7 out of 10 respondents in this group (1172/1669) self-report having no chronic diseases.

The second group, traditional self-trackers represents 25.58% (1051/1669) of our sample and 38.64% of all self-trackers (1051/2720). Unlike digital self-trackers, this group comprises slightly more women than men. The majority of traditional trackers are aged 55 years and older, retired, with a gross family income substantially less than digital trackers. Importantly, individuals in this group are more likely to be living with one or several chronic diseases than digital self-trackers (47.07% vs 28.36%, respectively).

The third group, nontrackers, represents 33.80% of our sample (1389/4109). Like traditional trackers, this group comprises slightly less men than women. However, nontrackers are found in all age groups. They are less educated and have lower gross family income on average than the other two groups. One in four nontrackers reported having one or several chronic conditions. The most common reasons given by these respondents for not tracking any aspect of their health were as follows: “the information provided by my physician is sufficient” (335/1389, 24.12%), “no need because I am in excellent health condition” (338/1389, 24.33%), “I am simply not interested” (329/1389, 23.69%), and “I am not disciplined enough” (261/1389, 18.79%). Four out of five nontrackers (1095/1389, 78.83%) possess a mobile phone or a tablet.

A multinomial logistic regression including all sociodemographic and health status variables was performed to calculate odds ratios describing the odds of tracking one’s own health using traditional or digital devices compared with the odds of nontracking (reference category). The traditional 0.05 criterion of statistical significance was employed for all tests. Addition of the predictors to a model that contained only the intercept significantly improved the fit between model and data; χ^2^_36_ (N=4109)=548.70, Nagelkerke *R*^2^=0.32, *P*<.001. As indicated in Table 3, our analyses determined no statistically significant differences between groups in terms of gender, region, occupation, and perceived health condition. However, significant differences were observed in terms of age, gross annual income, education level, and chronic condition(s). For instance, millennials (18-34 years) and people in the age range of 35 to 54 years were 3.7 and 1.5 times more likely to be in the digital self-tracking group than baby boomers (55+ years). As another example, compared with people living with no chronic condition, chronic patients were 0.4 times less likely to be in the traditional self-tracking group and 0.6 times less likely to be in the digital self-tracking group.

**Table 2:** Profile of self-trackers and nontrackers (N=4109).

Characteristics		Nontrackers (N=1389), n (%)	Traditional self-trackers (N=1051), n (%)	Digital self-trackers (N=1669), n (%)
**Gender**				
	Male	721 (51.91)	566 (53.85)	831 (49.79)
	Female	668 (48.09)	485 (46.15)	838 (50.21)
**Age in years**				
	18-34	314 (22.61)	147 (13.98)	684 (40.98)
	35-54	539 (38.80)	347 (33.02)	633 (37.93)
	55+	536 (38.59)	557 (53.00)	352 (21.09)
**Region**				
	Atlantic provinces	106 (7.63)	78 (7.42)	109 (6.53)
	Quebec	368 (26.49)	253 (24.07)	365 (21.87)
	Ontario	513 (36.93)	414 (39.39)	648 (38.83)
	Manitoba and Saskatchewan	94 (6.77)	65 (6.18)	107 (6.41)
	Alberta	124 (8.93)	103 (9.80)	211 (12.64)
	British Columbia and Terrace	184 (13.25)	138 (13.13)	229 (13.72)
**Gross family income ($ CAD)**				
	<$40k	335 (29.13)	244 (27.23)	272 (19.86)
	≥$40k and <$60k	244 (21.22)	171 (19.08)	200 (13.87)
	≥$60k and <$80k	190 (16.52)	154 (17.19)	216 (14.98)
	≥$80k and <$100k	145 (12.61)	109 (12.16)	244 (16.92)
	≥$100k and <$200k	195 (16.96)	192 (21.43)	428 (29.68)
	≥$200k	41 (3.56)	26 (2.90)	82 (5.69)
**Education level**				
	High school or college	805 (59.59)	529 (51.30)	717 (44.04)
	Undergraduate	376 (27.83)	330 (32.01)	593 (36.43)
	Graduate	170 (12.58)	172 (16.68)	318 (19.53)
**Occupation**				
	Workers	752 (54.85)	476 (45.81)	1158 (70.44)
	Students	53 (3.87)	23 (2.21)	75 (4.56)
	Retirees	347 (25.31)	383 (36.86)	207 (12.59)
	Other	219 (15.97)	157 (15.11)	204 (12.41)
**Perceived health condition**				
	Bad or average	118 (8.50)	127 (12.08)	157 (9.41)
	Good	712 (51.26)	524 (49.86)	833 (49.91)
	Very good or excellent	559 (40.24)	400 (38.06)	679 (40.68)
**Chronic diseases**				
	No	1021 (75.29)	542 (52.93)	1172 (71.64)
	Yes	335 (24.71)	482 (47.07)	464 (28.36)

**Table 3:** Multinomial logistic regression model predicting traditional tracking and e-tracking by patient characteristics. Reference category=nontrackers (N=1389).

Characteristics		Traditional self-trackers (N=1051)		Digital self-trackers (N=1669)	
		Odds ratio (95% CI)	Significance	Odds ratio (95% CI)	Significance
Intercept		—	<.001	—	<.001
**Gender**					
	Female	0.932 (0.765-1.134)	.48	1.170 (0.981-1.394)	.08
**Age (years)**					
	18-34	0.612 (0.434-0.863)	.005	3.732 (2.785-5.002)	<.001
	35-54	0.728 (0.555-0.954)	.02	1.552 (1.193-2.018)	<.001
**Region**					
	Atlantic provinces	1.055 (0.682-1.633)	.81	0.921 (0.619-1.370)	.69
	Quebec	1.022 (0.739-1.414)	.90	0.858 (0.641-1.147)	.30
	Ontario	1.038 (0.764-1.410)	.81	0.963 (0.733-1.263)	.78
	Manitoba-Saskatchewan	0.701 (0.434-1.134)	.15	0.859 (0.573-1.287)	.46
	Alberta	1.364 (0.900-2.067)	.15	1.335 (0.927-1.922)	.12
**Gross family income ($ CAD)**					
	≤$60K	0.750 (0.552-1.019)	.07	0.429 (0.326-0.566)	<.001
	> $60K and ≤ $100K	0.853 (0.673-1.081)	.19	0.550 (0.447-0.675)	<.001
**Education level**					
	High school or college	0.639 (0.477-0.855)	.003	0.623 (0.480-0.808)	<.001
	Undergraduate	0.797 (0.590-1.077)	.14	0.832 (0.637-1.086)	.18
**Occupation**					
	Workers	0.808 (0.608-1.075)	.14	1.292 (0.956-1.746)	.01
	Students	0.900 (0.442-1.834)	.77	0.680 (0.376-1.229)	.20
	Others	0.704 (0.483-1.027)	.07	0.877 (0.603-1.275)	.49
**Perceived health condition**					
	Very poor or poor	0.972 (0.669-1.414)	.88	1.108 (0.789-1.557)	.55
	Fair or good	0.923 (0.749-1.137)	.45	1.007 (0.837-1.212)	.94
**Chronic disease(s)**					
	One or several chronic disease(s)	0.403 (0.322-0.503)	<.001	0.548 (0.443-0.677)	<.001

### Motivations for Using Digital Self-Tracking Devices

The use of digital self-tracking devices and mobile apps is mainly the result of motivations tied to sustaining individual well-being rather than monitoring or mediating medical problems or illnesses. More precisely, 57.94% of our respondents (967/1669) said they use connected care technologies mainly to know more about their condition and monitor changes in parameters that they consider important for their health. Another common motivation was associated with the day-to-day encouragement that digital health self-tracking technologies provide as people strive to meet their personal goals (883/1669, 52.91%). Importantly, 42.06% (702/1669) said they use digital self-tracking tools to monitor their progress in fitness or athletic training. For their part, motivations such as “follow the treatment plan prescribed by my physician” (447/1669, 26.78%), “improve communication with my physician” (400/1669, 23.97%), and “reduce the number of medical visits” (381/1669, 22.83%) were not the primary drivers of use. Quite conversely, traditional self-trackers tend to monitor specific clinical parameters related to chronic conditions such as weight, heart rate, glucose level, and medication intake (see Table 4).

**Table 4:** Health aspects monitored by digital and traditional self-trackers.

Dimension and health aspects		Digital self-trackers (N=1669), n (%)	Traditional self-trackers (N=1051), n (%)
**Well-being**				
	Physical activity	856 (51.13)	441 (41.96)
	Nutrition and eating habits	545 (32.65)	392 (37.30)
	Sleep patterns	482 (28.88)	320 (30.45)
	Performance in sports	256 (15.34)	59 (5.61)
**Medical**				
	Weight-related data	483 (28.94)	585 (55.66)
	Cardiovascular and respiratory health (eg, heart rate)	215 (12.88)	300 (28.54)
	Medication intake	126 (7.55)	339 (32.25)
	Glucose level	79 (4.73)	247 (23.50)

### Adoption and Use of Wearable Connected Care Technologies

At the time of our survey, 74% of respondents (or 86% of those with a mobile phone or tablet) had already heard of consumer health wearables and smart medical devices. However, the level of familiarity with these remains relatively low, as only 16% of respondents who had already heard of such devices also reported being “very or extremely” familiar with them. We found that the level of familiarity with these tools is negatively correlated with age (*r*=−.21; *P*<.001) and positively correlated with family income (*r*=.14; *P*<.001). Indeed, millennials (χ^2^_4_=50.0; *P*<.001) and people with annual family income over $80,000 CAD (χ^2^_4_=28.2; *P*=.03) were more likely to be familiar with digital self-tracking devices than the other groups.

More importantly, our findings indicate that 1014 out of 1669 digital self-trackers (60.75%) own one or several wearables or smart medical devices, representing 24.68% of the entire sample. This ratio is similar to recent statistics from the United States. Indeed, according to two 2016 market reports, between 21% and 27% of American adults owned at least one such device [13,14]. Among our survey respondents, 70.02% (710/1014) said they had one connected wearable device, 20.71% (210/1014) had two, and 9.07% (92/1014) had three or more. On average, Canadian adults were using 1.5 consumer health wearable or smart medical device in early 2017. As these products are relatively new on the market, and our field administration shortly followed the 2016 holiday season, it is not surprising to observe that a majority of owners (549/1014, 54.14%) had been using their devices for less than a year at the time of the survey.

Although 61.11% of digital self-trackers (1020/1669) said they owned one or several wearable connected devices, 34.75% (580/1669) actually use them to self-track one or several aspects of their health. A multinomial logistic regression was performed to model the relationship between the predictors and membership in the two groups (nonusers and users of smart wearables). Addition of the predictors to a model that contained only the intercept significantly improved the fit between model and data; χ^2^_18_ (N=4109)=154.82, Nagelkerke *R*^2^=0.23, *P*<.05. As shown in Table 5, our analyses determined no statistically significant differences between users and nonusers of smart wearables in terms of gender, region, occupation, and education level. However, significant differences were observed in terms of age, gross annual income, perceived health status, and chronic condition(s). For one thing, millennials and people in the age range of 35 to 54 years were 2.2 and 1.6 times more likely to use digital devices to self-track their health than baby boomers (55+ years). For their part, people with annual family income inferior to $60,000 CAD were 0.38 times less likely to use smart digital devices than those with annual incomes of $100,000 CAD or more. Finally, our findings indicate that it is people who perceive themselves to be in very good or excellent health condition and those with no chronic condition who are current users of digital self-tracking devices.

In terms of usage, the most popular device is by far the bracelet or smartwatch, which is owned by 87.2% of those who own at least one such device (see Table 6). The main advantage often associated with wrist-wearable trackers is that they can monitor a range of health parameters and align with the common practice of wearing a watch. The bathroom scale and pedometer were the next most common connected devices used by Canadians.

Respondents were also asked how often they use connected self-tracking devices. Answers to this question varied across devices and according to the users’ specific needs. For instance, 77.5% of those who have a bracelet or a smartwatch (392/506) use it several times per day. For its part, the bathroom scale is generally used once a day (38/119, 31.9%) or several times per week (41/119, 34.5%), whereas a minority (13/119, 10.9%) use it only a few times per month. As a final example, the blood pressure monitor is used once a month (12/47, 25%), once a day (11/47, 24%), or a few times per day (8/47, 16%) depending on the individual’s condition and needs.

**Table 5:** Multinomial logistic regression model predicting usage of health wearables and smart medical devices by patient characteristics. Reference category=nonusers (N=3529).

Characteristics		Users of health wearables and smart medical devices (N=580)	
		Odds ratio (95% CI)	Significance
Intercept		—	<.001
**Gender**			
	Female	1.041 (0.846-1.282)	.70
**Age (years)**			
	18-34	2.234 (1.577-3.167)	<.001
	35-54	1.566 (1.128-2.174)	.007
**Region**			
	Atlantic provinces	0.962 (0.592-1.563)	.88
	Quebec	0.752 (0.522-1.083)	.13
	Ontario	1.120 (0.811-1.546)	.49
	Manitoba-Saskatchewan	0.993 (0.605-1.629)	.98
	Alberta	1.242 (0.834-1.850)	.29
**Gross family income ($ CAD)**			
	≤60K	0.381 (0.262-0.554)	<.001
	>60K and ≤100K	0.638 (0.511-0.797)	<.001
**Education level**			
	High school or college	0.861 (0.644-1.152)	.31
	Undergraduate	1.071 (0.809-1.419)	.63
**Occupation**			
	Workers	1.255 (0.859-1.833)	.24
	Students	0.377 (0.146-0.975)	.04
	Others	0.780 (0.471-1.292)	.34
**Perceived health status**			
	Very poor or poor	0.428 (0.267-0.685)	<.001
	Fair or good	0.689 (0.556-0.854)	<.001
**Chronic disease(s)**			
	One or more chronic condition(s)	0.784 (0.615-0.998)	.049

### Data Sharing With Health Care Providers

This study indicates that there are relatively few people who regularly share the data captured with their digital self-tracking devices. In fact, only 34.87% of users (582/1669) reported that they share their personal health data. When they do so, it is primarily with family members (352/582, 60.5%), friends (294/582, 50.5%), and to a much lesser extent, an HCP such as a family doctor (195/582, 33.5%) or a pharmacist (50/582, 8.6%). Although no direct comparisons could be made with other surveys, empirical evidence in the United States shows that 78% of adults who use health wearables would like their doctor to have access to their personal data [14]. Another recent survey conducted in Canada reveals that 67% of users of mobile apps would share their data if their doctor requested it [24].

### Users’ Appreciation of Connected Care Technologies

As shown in Tables 7 and 8, users of consumer wearables and connected devices claimed to be very satisfied (mean=4.1 on a 5-point Likert scale), perceived their devices to be user-friendly (mean=4.2), and had a firm intention of continuing to use them in the future (mean=4.3). Overall, respondents perceive these devices as highly useful. About 7 out of 10 users (398/580) said that they have maintained or improved their health status by using digital self-tracking connected devices. Importantly, a majority of users report they are more informed or more knowledgeable about their health condition. Close to 6 out of 10 users (435/580) said they feel more confident taking care of their health or more autonomous in the management of their condition. On the other hand, feeling less anxious about one’s own health and having more informed discussions with a doctor were not perceived as major benefits digital self-trackers in our study.

**Table 6:** Types of consumer wearables and smart medical devices among Canadian adults who use at least one such device (N=580).

Types of wearables	n (%)
Bracelet, wristband or smartwatch	506 (87.2)
Bathroom scale	119 (20.5)
Pedometer	76 (13.1)
Blood pressure monitor	47 (8.1)
Intelligent toothbrush	38 (6.6)
Pulse oximeter or spirometer (respiratory functions)	35 (6.0)
Thermometer	33 (5.7)
Glucose monitor	25 (4.3)
Intelligent clothes (eg, pants, shirts, and socks)	20 (3.4)
Spirometer	16 (2.8)
Intelligent pill dispenser	14 (2.4)
Intelligent fork	11 (1.9)

**Figure 1:**
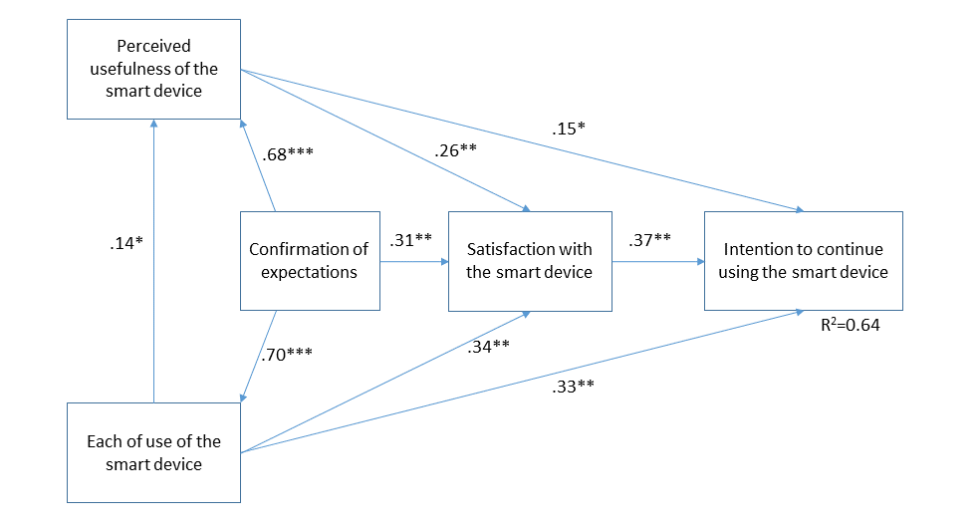
Users’ appreciation of smart devices (N=580); ***P<.005; **P<.01; *P<.05.

To further investigate users’ appreciation of digital self-tracking devices, we tested a research model derived from the works of Bhattacherjee [20] and Hong et al [21], as well as expectation-confirmation theory [25]. To our knowledge, no prior research has investigated the factors influencing the continued usage of these devices. As shown in Figure 1, our model suggests that an individual’s intention to continue using health wearables and smart devices is mainly influenced by his or her level of satisfaction. In turn, user satisfaction is influenced by the extent to which initial expectations toward these devices are confirmed, as well as by two factors from the Technology Acceptance Model (TAM) proposed by Davis [19], namely, ease of use and perceived usefulness. Following Hong et al [21], our model also proposes direct links between the TAM constructs and the dependent variable.

The reliability of the measures included in the model was determined with Cronbach alpha. Findings in Table 6 indicate that all the measures, without exception, surpass the 0.70 threshold of statistical significance [26]. This table also demonstrates the validity of the variables included in our research model. In particular, we see that the square root of the variance shared by each variable and its respective items is greater than the intercorrelations between the variables.

PLS regression analyses were performed to test the links in the model. Our findings in Figure 1 supported all relationships, and the model explains 64% of the variance in the dependent variable. Our results indicate that expectations confirmation is strongly related to TAM factors and user satisfaction. This result shows the importance of properly managing consumers’ initial expectations to ensure greater adherence and continued usage of health wearables and smart medical devices. Future research on this topic may consider other variables such as information quality and personalization of content that have been recognized as facilitators for adherence (eg, [27]).

### Reasons for Abandoning the Use of Digital Self-Tracking Devices

A 2015 study [28] suggested that one-third of consumer wearables end up in a drawer 6 months after purchase or initial use. We observed a slightly reduced observation of this phenomenon, with 25.54% of owners (259/1014) who had stopped using their connected devices at the time of the survey. When asked “Why did you stop using your device(s)?” a majority of respondents (111/259, 42.9%) said they had “lost interest after a while.” Other reasons included malfunctioning of the device (51/259, 19.7%), doubts about the reliability of the data (39/259; 15.1%), and “the device was acquired more out of curiosity” (38/259, 14.7%). Most interestingly, we found that while abandoning use of these devices was not associated with gender, age, region, education, or main occupation, it was more prevalent among those who perceive there current health status as “poor or fair” compared with those who self-report their health status as good or excellent (χ^2^_4_=6.6; *P=*.048).

### Reasons for Not Owning Digital Self-Tracking Devices

Respondents who do not own consumer health wearables or connected devices (n=2035) were asked why. Our results indicate that 46.93% of this segment (955/2035) did not see the interest in owning such tools. Other obstacles to greater diffusion of digital self-tracking devices were related to cost (836/2035, 41.08%), lack of knowledge about the value or benefits associated with the use of these devices (368/2035, 18.08%), and doubts about the reliability of data (341/2035, 16.76%). As many nonowners have limited knowledge of the value proposition for such devices, it is not surprising to observe that intentions to buy and adopt one in the near future were relatively low. Indeed, slightly less than 15% of nonowners (14.99%, 305/2035) reported that they intend to acquire a health wearable or smart medical device in the next 12 months.

**Table 7:** Users’ appreciation of connected care technologies.

Variable and items		Somewhat or strongly disagree, n (%)	Neutral, n (%)	Somewhat or strongly agree, n (%)
**Perceived usefulness**				
	I have maintained or improved my health condition	31 (5.4)	151 (26.1)	398 (68.5)
	I am more informed about my health	47 (8.1)	147 (25.1)	387 (66.6)
	My knowledge of my health condition has improved	51 (8.8)	179 (30.9)	350 (60.3)
	I feel more confident taking care of my health	51 (8.8)	194 (33.5)	435 (57.7)
	I am more autonomous in the management of my health	37 (6.4)	215 (37.1)	328 (56.5)
	I feel less anxious about my health	81 (14.1)	239 (41.2)	259 (44.8)
	I have more informed discussions with my doctor	94 (16.1)	249 (42.9)	238 (41.0)
**User friendliness**				
	I find it easy to use my wearables or smart devices	18 (3.1)	57 (9.8)	506 (87.1)
	I find my wearables or smart devices user-friendly	22 (3.9)	58 (9.9)	500 (86.2)
	Learning how to use my wearables or smart devices was easy	28 (4.9)	65 (11.3)	486 (83.9)
	The information provided stored in the mobile apps is easy to understand and interpret	29 (5.0)	57 (9.9)	493 (85.1)
**User satisfaction**				
	I am satisfied with the use of my wearables or smart devices	28 (4.8)	71 (12.2)	481 (83.0)
	I am pleased with the use of my wearables or smart devices	28 (4.8)	71 (12.2)	481 (83.0)
	I am delighted with the use of my wearables or smart devices	25 (4.4)	114 (19.6)	441 (76.0)
**Confirmation of initial expectations**				
	My initial expectations concerning my use of wearables or smart devices have been confirmed so far	26 (4.6)	109 (18.7)	445 (76.7)
	Using my wearables or smart devices turned out to be easier that I first thought	36 (6.2)	141 (24.3)	404 (69.5)
	There are more benefits to using my wearables or smart devices than I first thought	42 (7.3)	150 (25.8)	388 (66.8)
**Intention to continue using**				
	I have every intention of continuing to use wearables or smart devices in the future	23 (4.0)	45 (7.8)	511 (88.2)
	I will continue to use wearables or smart devices to monitor different aspects of my health	19 (3.2)	70 (12.0)	492 (84.7)
	I have no intention of stopping my use of wearables or smart devices in the future	22 (3.9)	64 (11.1)	493 (85.1)

**Table 8:** Descriptive statistics and variance shared by the variables (N=580). The ratios in italics on the diagonal represent the square root of the variance shared by each variable and its respective items. The ratios above the diagonal are Pearson correlation coefficients between variables.

Variables	Mean (SD); 1-5	Number of items	Cronbach alpha	Perceived usefulness	Ease of use	Confirmation of initial expectations	User satisfaction	Intention to continue usage
Perceived usefulness	3.6 (0.7)	7	.90	*.80*	.53^a^	.77^a^	.66^a^	.56^a^
Ease of use	4.2 (0.7)	4	.92	—	*.90*	.71^a^	.73^a^	.74^a^
Confirmation of initial expectations	3.9 (0.7)	3	.80	—	—	*.84*	.77^a^	.67^a^
User satisfaction	4.1 (0.8)	3	.89	—	—	—	*.90*	.70^a^
Intention to continue usage	4.3 (0.8)	3	.91	—	—	—	—	*.92*

^a^*P*<.001.

## Discussion

### Strengths and Limitations

This study investigates Canadian adults’ digital health self-tracking behaviors and their use of connected wearables and devices to monitor aspects of their health and well-being. To our knowledge, it is one of the most comprehensive studies on this topic, resulting in highly reliable estimates of findings. Hence, our results set important baseline information that will guide future research on the evolution of the quantified-self phenomenon. Importantly, these findings are relevant to the information and technology industry and mHealth app developers to better understand the current market, segments, and viabilities to achieve behavioral and clinical outcomes. We further contributed to the extant literature by investigating novel, yet important aspects and issues, including the reasons for self-tracking and nontracking, the barriers to adoption of digital devices, consumers’ appreciation of wearables and smart devices, the perceived benefits associated with digital self-tracking, and the reasons for usage discontinuance. Hence, this work may inform future policies and efforts in relation to general incorporation of self-tracking digital devices as supportive tools for patient care or reimbursement for technology-enabled quality outcome models of care.

Notwithstanding these strengths and contributions, our results must be interpreted with caution because of some inherent limitations. First, the responses relied on self-report and included only people who participate in Web panels managed by the survey company. Second, this is a cross-sectional survey, and while helpful for examining self-tracking behaviors and use of connected care technologies at one point in time, it is likely that people vary their use patterns and behaviors over time. Third, we did not collect data about race and ethnicity, although these variables might be related to the use of connected health technologies. Finally, our survey did not include people’s health literacy, which may represent an important moderator.

### Implications for Practice and Research

Our survey first reveals that the digital health self-tracking movement in Canada is still in an early stage. About one in 4 respondents currently owns a health wearable or smart medical device. Among them, 57.20% (580/1014) use their smart devices on a regular basis for self-tracking purposes. Digital health self-trackers are mainly young, highly educated, and wealthy individuals whose main motivation for use of connected technologies is to monitory or quantify their fitness behaviors or progress on fitness goals. These results indicate an important presence of a significantly health engaged and activated segment of the Canadian population. Indeed, our findings show that many Canadian adults self-track aspects of their health because of the ubiquitous nature of mobile apps for health and consumer wearables and connected devices.

Although the use of connected care technologies could potentially benefit the growing population of patients with chronic conditions [29,30], the question remains as to whether it will diffuse broadly beyond early adopters and across cost inequities. Although technology manufacturers may assume patients with chronic conditions have unlimited enthusiasm for tracking their own health data using self-tracking devices, reality seems to be otherwise. Indeed, our findings show that 29.3% of those with chronic conditions in our sample had abandoned the use of their devices at some point compared with 13.8% for those with no chronic diseases (*P=*.04). A plausible explanation may be that chronic patients often consider it work (ie, a consuming and tiring task) to track their own health data [31]. This would suggest that digital self-tracking devices will successfully spread among chronic patients only if they are highly activated as patients and use is not experienced as a burden on the user but a positive and rewarding user experience. The use of gamification and positive reinforcement techniques [32,33] may represent effective ways of making the experience more enjoyable and useful for chronic patients. Another explanation may be associated with the fact that medical parameters being tracked by chronic patients can be emotionally charged [31]. Indeed, “bad” data values can be extremely upsetting for many patients, especially when they are perceived to have some link to behavior. A third explanation may be that HCPs, especially physicians, do not seem interested in patients’ self-logged data—even data that may be entirely objectively logged [31]. We believe chronic patients, especially those with severe conditions, may need personal coaching and continued support from HCPs to ensure adherence, system continuance, and positive health outcomes. This recommendation is also aligned with the importance of properly managing users’ expectations discussed earlier.

To deepen our understanding of usage discontinuance, a prevalent phenomenon identified in these results, we suggest that future research include an approach that borrows a model proposed by Li et al [7]. This model outlines five psychological stages in the process of engaging in digital self-tracking. The first stage, called preparation, concerns people’s motivation to collect personal information, how they determine what information to collect, and how they will record it. The next step, collection, is when people collect information about themselves. Integration is the stage where the information collected is prepared, combined, or transformed for the use to reflect on (reflection stage). Finally, action is when people choose what they are going to do with their newfound understanding of themselves (eg, people may tailor their behaviors to match their goals). A key finding of Li et al’s study is that individuals have a tendency to focus on a single stage (ie, collecting data on number of steps or hours slept) and to ignore the overall process and intended outcome of self-tracking for health outcomes. This reinforces the importance of providing professional coaching and continued support. Among others, future studies could use this model to identify the barriers that people, especially chronic patients, experience when they self-track using connected care technologies.

Although 70% of digital self-trackers in our sample feel they have maintained or improved their health by using wearables and smart devices, there is little empirical evidence that suggest self-tracking personal health indicators leads to long-term behavioral changes [34]. Among the few studies we found, one trial concludes that the use of pedometers along with nursing consultations increased physical activity among older adults [35]. In another trial, the use of a wearable tracker by overweight and obese adults led to a small increase in moderate-to-vigorous intensity physical activity at follow-up [36]. More research with large samples is definitely needed to determine the effectiveness of wearable connected devices on people’s physical activity. It is also unclear whether such devices can motivate adults of all age groups toward other important health mediating behaviors such as adopting a healthy diet, maintaining a healthy weight, adopting good sleep habits, and not smoking. Because healthy behaviors will lead to significant improvements in population health only if they are sustained [37], it will also be important that future (longitudinal) trials investigate whether and under which conditions (eg, health literacy) digital health self-tracking devices can support the creation and maintenance of enduring new lifestyle habits and improve quality of life.

Finally, prior research shows that connected care technologies may create new opportunities for individuals who desire to participate actively in and take responsibility for their personal health. As discussed in Kitsiou et al [38], mobile apps along with consumer wearables and smart medical devices can provide a platform for home telemonitoring programs for chronic patients. Furthermore, physicians can use wearable sensors to monitor acute patients’ health in real time, which can aid with diagnosis and treatment decisions [34]. For instance, chronic sleep apnea can be diagnosed with a lightweight wearable that measures heart rate, breathing volume, and snoring instead of a heavy polysomnography assessment [39]. In addition, incorporating wearable and smart device sensors into routine care may improve clinician-patient relationships and increase patient empowerment [40]. It also appears that the widespread integration of these devices into medical practice by clinicians is extremely limited [41]. A recent survey of 989 Canadian HCPs shows that only 30% recommend wearable trackers (eg, smartwatch and bracelet) to their patients, and 25% recommend medical smart devices such as blood pressure monitors and sleep trackers [42]. Several barriers related to patient safety, data accuracy and security, reimbursement policy, and government regulation have been discussed in the extant literature (eg, [29,43]). Future research must continue investigating these important issues for practicing clinicians so that we develop a better understanding of how and under which circumstances the use of connected care technologies can best serve medicine, in general, and prevention and management of chronic conditions, in particular.

## Appendix

Multimedia Appendix 1 Survey instrument.

## Abbreviations

HCP: health care provider
mHealth: mobile health
PLS: partial least squares
TAM: Technology Acceptance Model
