# Supplementary material for: Diffusion of the Digital Health Self-Tracking Movement in Canada: Results of a National Survey
Source: J Med Internet Res. 2018 May 2;20(5):e177. doi: 10.2196/jmir.9388 (PMC5956159; doi:10.2196/jmir.9388)
Supplement: Multimedia Appendix 1 [file jmir_v20i5e177_app1.pdf]

Multimedia Appendix. Survey Instrument

Section 1. Background

Q1a. Do you own any of the following mobile devices?

	Yes	No
a) A smartphone that can be used to download mobile applications (apps)* (for example: Apple iPhone, Samsung Galaxy, Google Nexus, Microsoft Lumia, Sony Xperia)	1 ☐	0 ☐
b) A digital tablet that can be used to download mobile applications (apps)* (for example: Apple iPad, Samsung Galaxy Tablet, Google Nexus Tablet, Sony Xperia Tablet)	1 ☐	0 ☐

IF THE ANSWERS TO a) AND b) ARE BOTH 0, GO TO Q13a, OTHERWISE CONTINUE

**A mobile*

Q1b. Generally speaking, how often do you access the Internet using your smartphone and/or digital tablet, for example to read the news, go on Facebook, check the weather forecast or listen to the radio?

Many times each day	7 ☐
A few times each day	6 ☐
Once a day	5 ☐
3 to 5 times per week	4 ☐
1 to 2 times per week	3 ☐
2 to 3 times per month	2 ☐
Once a month or less	1 ☐
I never access the Internet on my mobile device(s)	99 ☐

CONTINUE

Section 2. Mobile Apps

Q2. Do you have one or more mobile apps on your smartphone or digital tablet to help you monitor certain aspects of your health or well-being (e.g. your weight, your dietary habits, the quality of your sleep, your mood, your physical activity, your blood pressure, your blood sugar level)?

Yes	1 ☐	**CONTINUE**
No	0 ☐	**GO TO Q13a**

Q3. Have you, in the last 3 months, used at least one health or well-being mobile app?

Yes	1 ☐	**GO TO Q5**
No	0 ☐	**CONTINUE**

PRIMARY STUDY SAMPLE CRITERIA: ANSWWERS TO Q3 = 1 'YES'; and/or Q13C= 1 'YES, and I use them' or 2 'YES, but I have stopped using them' . Sample Completes (Target) N = 1000

Q4. Indicate the reason or reasons why you have not used this type of mobile app in the last three months. **Please check all the boxes that apply to your personal situation.**

**RANDOM ROTATION EXCEPT FOR ITEM "M," WHICH SHOULD ALWAYS BE LAST**	**Checked=1; otherwise=0**
a) Entering data (e.g. on weight, distance covered, blood sugar level) in an app is too time-consuming.	☐
b) At one point I found that I wasn't learning anything new.	☐
c) There were hidden costs associated with using the app.	☐
d) The app was too complicated to use.	☐
e) I had doubts about the reliability of the information generated by the app.	☐
f) I wasn't able to reach my goals and lost interest.	☐
g) I didn't like the idea of sharing my personal information with other people.	☐
h) I was worried my data would be transmitted without my permission/consent.	☐
i) I was worried that unauthorized third parties would make inappropriate use of my personal data.	☐
j) I was worried that using these apps could become an obsession.	☐
k) After a while, I just lost interest in this type of app.	☐
l) The app that I was using just stopped working well.	☐
m) No specific reason.	☐

GO TO Q13a

Q5. **In total,** how many health or well-being mobile apps have you used <u>in the last 3 months</u>?

1	1 ☐
2	2 ☐
3	3 ☐
4	4 ☐
5	5 ☐
6	6 ☐
7	7 ☐
8	8 ☐
9	9 ☐
10 or more	10 ☐
I don’t know	99 ☐

CONTINUE

Q6. How long have you been using these apps?

Less than 3 months	1 ☐
Between 3 and 6 months	2 ☐
Between 6 and 12 months	3 ☐
Between 1 and 2 years	4 ☐
Between 2 and 5 years	5 ☐
I don’t remember exactly	99 ☐

CONTINUE

Q7. Concerning your health and well-being, which of the following items do the apps you currently use help you with …?
Please check all the boxes that apply to your personal situation.

**RANDOM ROTATION**	**Checked=1; otherwise=0**
**a)** Competition and performance in sports<br>For example: training guides, as a record of sports performance; calculations of distance covered or calories burned.	☐
**b)** Regular physical activity<br>For example: exercise guides/routines; advice on leading a physically active life (being more active); as a record of physical activity; step counter, calories burned.	☐
**c)** Nutrition and eating habits<br>For example: guides/programs/tools for balanced nutrition; meal calorie calculator.	☐
**d)** Weight-related information<br>For example: Monitoring weight or waistline; calculator of body mass index.	☐
**e)** Sleep<br>For example: monitoring sleep quality and/or hours slept; advice/tools for better sleep – music, alarms, etc.; monitoring sleep conditions, such as snoring or sleep apnea.	☐
**f)** Cardiovascular, lung or respiratory airway health<br>For example: tools/advice for monitoring blood pressure, heart rate, pulse, asthma, oxygen levels	☐
**g)** Diabetes and other metabolism-related conditions<br>For example: sugar, cholesterol	☐
**h)** Use of medication<br>For example: monitoring medication use; identifying side effects or contraindications	☐
**i)** Sexual and reproductive health<br>For example: Women: menstrual cycle; guides/advice on monitoring a pregnancy or the postnatal period. Men: guides/advice for sexual health	☐
**j)** Mental and emotional health<br>For example: monitoring mood/emotional state; stress management; guides/tools for meditation/relaxation or motivation; monitoring/guides/tools for memory, attention, cognitive skills	☐
**k)** Dental health	☐
**l)** Tobacco dependence<br>For example: monitoring/guides/tools for reducing or ending tobacco consumption	☐
**m)** Alcohol and drugs<br>For example: monitoring/guides/tools for monitoring goals to reduce alcohol intake, support harm reduction or abstinence strategies to reduce or end alcohol or drug consumption	☐

FOR EACH ITEM CHECKED IN Q7, ASK Q8 [Single Question per page, with Q8 Prompted if item =1]

Q8. How often do you update your data on this aspect of health or well-being using your mobile app(s)?

Many times each day	7 ☐
A few times each day	6 ☐
Once a day	5 ☐
3 to 5 times per week	4 ☐
1 to 2 times per week	3 ☐
2 to 3 times per month	2 ☐
Once a month or less	1 ☐

CONTINUE

Q9. Generally speaking, to what extent do each of the following items encourage you to use one or more apps to better monitor your health or well-being?

RANDOM ROTATION	5 Very strongly	4 Rather strongly	3 Somewhat	2 Mildly	1 Not at all
a) Know myself better and monitor changes in things that I consider important for my health (e.g. weight, physical activity, sleep, etc.)	☐	☐	☐	☐	☐
b) Break a bad habit related to my health	☐	☐	☐	☐	☐
c) Give me daily encouragement toward reaching my personal health and wellness goals	☐	☐	☐	☐	☐
d) Monitor progress made in my athletic training	☐	☐	☐	☐	☐
e) Better follow the treatment plan prescribed by my physician or another health professional	☐	☐	☐	☐	☐
f) Monitor one or more issues related to one or more chronic illnesses (e.g. diabetes, high blood pressure, asthma, obesity)	☐	☐	☐	☐	☐
g) Maintain or improve my autonomy to live independently in my home (e.g. preparing meals, reminders for daily activities and routines, like grocery shopping))	☐	☐	☐	☐	☐
h) Help me take my medication on time as it was prescribed					
i) Reduce the number of times I need to see my doctor	☐	☐	☐	☐	☐
j) Improve communication with my physician or another health professional	☐	☐	☐	☐	☐

CONTINUE

Q10. Do you ever share the data on health and well-being recorded in your app(s) with other people?

Yes	1 ☐	CONTINUE
No	0 ☐	GO TO Q12
Prefer not to answer	88 ☐	GO TO Q12

Q11. With whom do you usually share the data on health and well-being recorded in your mobile apps? **Please check all the boxes that apply to your personal situation.**

RANDOM ROTATION EXCEPT FOR ITEM "G," WHICH SHOULD ALWAYS BE LAST	Checked=1;

	otherwise=0
a) Family members (e.g. spouse, brother/sister, parent, child)	☐
b) Friends	☐
c) My family doctor at my regular place of care	☐
d) A nurse at my regular place of care	☐
e) My pharmacist	☐
f) Nutritionist	☐
g) My counsellor or therapist supporting my mental health	☐
h) My personal trainer (coach)	☐
i) Other users of the same mobile app	☐
j) Individuals or groups on social media	☐
k) Someone else – please specify:	☐

CONTINUE

Q12. To what extent do you agree or disagree with each of the following statements?

RANDOM ROTATION	1 Strongly disagree	2 Somewhat disagree	3 Neutral	4 Somewhat agree	5 Strongly agree
a) I am satisfied with my use of apps	☐	☐	☐	☐	☐
b) I am pleased with my use of apps	☐	☐	☐	☐	☐
c) I am delighted with my use of apps	☐	☐	☐	☐	☐
d) Learning how to use my app(s) was easy	☐	☐	☐	☐	☐
e) I find my app(s) user-friendly	☐	☐	☐	☐	☐
f) The information provided by my app(s) is easy to understand and interpret	☐	☐	☐	☐	☐
g) In general, I find it easy to use my app(s)	☐	☐	☐	☐	☐
h) Using my app(s) turned out to be easier than I first thought	☐	☐	☐	☐	☐
i) There were more benefits to using my app(s) than I first thought	☐	☐	☐	☐	☐
j) My expectations concerning how I would use my app(s) have been confirmed so far	☐	☐	☐	☐	☐
k) Thanks to my app(s), I have learned to be better informed about my health	☐	☐	☐	☐	☐
l) My use of app(s) allows me to be more autonomous in the management of my health and well-being	☐	☐	☐	☐	☐
m) I have maintained or improved my health status by using apps	☐	☐	☐	☐	☐
n) Overall health apps have proved very useful in my life	☐	☐	☐	☐	☐
o) I have every intention of CONTINUING to use health app(s) in the future	☐	☐	☐	☐	☐
p) I have no intention of stopping my use of health app(s)	☐	☐	☐	☐	☐
q) I will CONTINUE to use apps to measure, on my own, different aspects of my health and well-being	☐	☐	☐	☐	☐
r) Because of my use of health apps, I feel more anxious about my health.	☐	☐	☐	☐	☐
s) Because of my use of health apps, I feel I can have more informed discussions with my doctor.	☐	☐	☐	☐	☐
t) Because of my use of health apps, I feel more confident taking care of my health	☐	☐	☐	☐	☐
u) Because of my use of health apps, my knowledge of my health has improved	☐	☐	☐	☐	☐

CONTINUE

Section 3. Health and Well-Being Smart Connected Devices

Q13a.

The following questions deal with smart connected devices that are used to monitor health and well-being. They are electronic objects that, like those shown below, capture data on different aspects of one's health and well-being, such as pulse, weight, athletic performance, sleep quality, body temperature and blood pressure, and synch via WiFi or bluetooth with an app on your mobile smartphone or digital tablet or plug in and synch directly with an application or program on your desktop computerfor visual display, monitoring, tracking, and/or analysis.

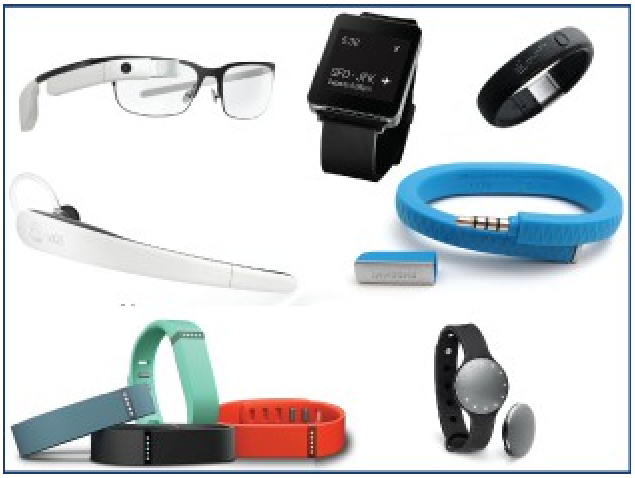

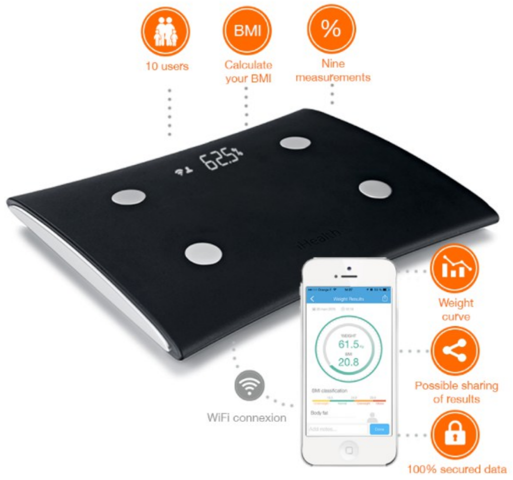

Before today, had you ever heard about smart devices for health and well-being?

Yes	1 ☐	CONTINUE
No	0 ☐	IF Q3 = 0, GO TO Q20, OTHERWISE GO TO Q24

Q13b. How familiar are you with smart devices for health and well-being?

Not much at all	Slightly	Somewhat	Very	Extremely
1 ☐	2 ☐	3 ☐	4 ☐	5 ☐

CONTINUE

Q13c. Do you have one or more smart devices or wearables for health and wellbeing that capture data related to your health and well-being?

YES, and I use them	1 ☐	**CONTINUE**
YES, but I have stopped using them	2 ☐	**GO TO Q13e**
YES, but I have never used them	3 ☐	**IF Q3 = 0, GO TO Q20, OTHERWISE GO TO Q24**
NO	4 ☐	**GO TO Q17**

Q13d. How long have you been using a smart device/wearable for health and well-being?

Less than 3 months	1 ☐
Between 3 and 6 months	2 ☐
Between 6 and 12 months	3 ☐
Between 1 and 2 years	4 ☐
Between 2 and 5 years	5 ☐
I don't really remember	99 ☐

GO TO Q14

13e. Why did you stop using the smart device you have? **Check all the reasons that apply to your personal situation.**

RANDOM ROTATION EXCEPT FOR ITEM "K," WHICH SHOULD ALWAYS BE LAST	Checked=1; otherwise=0
a) I found this type of object too complicated to use.	☐
b) I didn't like carrying or wearing this type of device with /on me.	☐
c) I wasn't able to attain the objectives I had set for myself, so I lost my motivation.	☐
d) This type of device didn't meet my personal expectations.	☐
e) I had acquired this type of device more out of curiosity than to make use of it.	☐
f) Capturing data with this type of device took too much of my time.	☐
g) The device(s) I had simply stopped working well.	☐
h) After a while, I just lost interest in this kind of device	☐
i) I had doubts about the reliability of the information generated by the device(s) I was using.	☐
j) For no particular reason.	☐
k) I didn't like taking the time to synch my device with the mobile app it came with	☐

GO TO QUESTION 24

Q14. How many smart devices for health and well-being do you currently own?

1	1 ☐
2	2 ☐
3	3 ☐
4	4 ☐
5	5 ☐
6	6 ☐
7	7 ☐
8	8 ☐
9	9 ☐
10 or more	10 ☐
I don't know	99 ☐

CONTINUE

Q15a. Which of the following smart devices for health and well-being do you own? **Please check all the boxes that apply to your personal situation.**

RANDOM ROTATION EXCEPT FOR ITEMS "L" and "M," WHICH ARE ALWAYS PLACED LAST	Checked=1; otherwise=0
a) Bracelet , wristband, or watch	☐
b) Intelligent clothing (e.g. pants, shirt, t-shirt, socks, hat, belt, shoe soles)	☐
c) Bathroom scale	☐
d) Toothbrush	☐
e) Fork *(eating speed, calories consumed)*	☐
f) Blood pressure monitor	☐
g) Pedometer *(steps walked or run)*	☐
h) Thermometer	☐
i) Glucose monitor	☐
j) Intelligent pill dispenser	☐
k) Pulse oximeter or spirometer *(respiratory functions)*	☐
l) Other connected/intelligent devices worn using a band (e.g. worn on the head, the neck, an arm, a thigh)	☐
m) Other portable connected objects (e.g. connected optical devices, connected pendants, connected hearing aids)	☐

FOR EACH OBJECT CHECKED IN Q15a, ASK Q15b [Single Question per page, with Q8 Prompted if item =1]

Q15b. How often do you use this smart device for health and well-being?

[DISPLAY THE ITEM CHECKED IN Q15a]

Many times each day	7 ☐
A few times each day	6 ☐
Once a day	5 ☐
3 to 5 times per week	4 ☐
1 to 2 times per week	3 ☐
2 to 3 times per month	2 ☐
Once a month or less	1 ☐

CONTINUE

Q16. Indicate the extent to which you agree or disagree with each of the following statements.

RANDOM ROTATION	1 Strongly disagree	2 Somewha t disagree	3 Neutral	4 Somewha t agree	5 Strongly agree
a) I am satisfied with the use I am making of my smart device(s) for heath	☐	☐	☐	☐	☐
b) I am pleased with the use I am making of my smart device(s) for heath	☐	☐	☐	☐	☐
c) I am delighted with the use I am making of my smart device(s) for heath connected object	☐	☐	☐	☐	☐
d) Learning how to use my smart device(s) for heath was easy	☐	☐	☐	☐	☐
e) I find my smart device(s) for heath user-friendly	☐	☐	☐	☐	☐
f) The information provided by my smart device(s) for heath is easy to understand and interpret	☐	☐	☐	☐	☐
g) In general, I find it easy to use my smart device(s) for heathobject(s)	☐	☐	☐	☐	☐
h) Using my smart device(s) for heathturned out to be easier than I first thought	☐	☐	☐	☐	☐
i) There were more benefits to using my smart device(s) for heaththan I first thought	☐	☐	☐	☐	☐
j) My expectations concerning how I would use my smart device(s) for heathhave been confirmed so far	☐	☐	☐	☐	☐
k) Thanks to my smart device(s) for heath, I have learned to be better informed about my health	☐	☐	☐	☐	☐
l) My use of smart device(s) for heathallows me to be more autonomous in the management of my health and well-being	☐	☐	☐	☐	☐
m) I have maintained or improved my health status by using smart device(s) for heath	☐	☐	☐	☐	☐
n) Overall, smart device(s) for heath have proven very useful in my life	☐	☐	☐	☐	☐
o) I have every intention of CONTINUING to use my smart device(s) for heath in the future	☐	☐	☐	☐	☐
p) I have no intention of stopping my use of smart device(s) for heath	☐	☐	☐	☐	☐
q) I will continue to use smart device(s) for heathto measure, on my own, different aspects of my health and well-being	☐	☐	☐	☐	☐
r) My use of smart device(s) for health, help me feel more anxious about my health.	☐	☐	☐	☐	☐
s) Because of my use of health apps, I feel I can have more informed discussions with my doctor.	☐	☐	☐	☐	☐
t) Because of my use of health apps, I feel more confident taking care of my health	☐	☐	☐	☐	☐
u) Because of my use of health apps, my knowledge of my health has improved	☐	☐	☐	☐	☐

GO TO Q24

Q17. For which of the following reasons do you not own smart devices for health and well-being? **Please check all the boxes that apply to your personal situation.**

RANDOM ROTATION EXCEPT FOR ITEM "K," WHICH SHOULD ALWAYS BE LAST	Checked=1; otherwise=0
a) I am not interested	☐
b) I do not know enough about the benefits of smart device(s) for heath.	☐
c) I'm worried that I won't know how to make good use of them.	☐
d) I have doubts about the reliability of the measures they take.	☐
e) I feel that they would intrude on my privacy.	☐
f) I am worried that unauthorized third parties will make inappropriate use of my personal data.	☐
g) I am worried that use of these smart devices will become an obsession and a source of concern.	☐
h) I am worried of becoming overly dependent on these devices.	☐
i) Smart device(s) for health are too expensive.	☐
j) My physician does not seem to think they are worthwhile or has not spoken to me about them.	☐
k) None of the above.	☐

CONTINUE

Q18. Are you thinking about buying a health and well-being connected object <u>in the next 12 months</u>?

Very likely	5 ☐	CONTINUE
Somewhat likely	4 ☐	CONTINUE
Unlikely	3 ☐	IF Q3 = 0, GO TO Q20, OTHERWISE GO TO Q24
Very unlikely	2 ☐	IF Q3 = 0, GO TO Q20, OTHERWISE GO TO Q24

Not at all likely	1 ☐	**IF Q3 = 0, GO TO Q20, OTHERWISE GO TO Q24**
Don’t know	99 ☐	**CONTINUE**

Q19. Which of the following smart devices for health would you be interested in acquiring in the next 12 months?
Please check all the boxes that apply to your situation.

RANDOM ROTATION EXCEPT FOR ITEMS “L” AND “M,” WHICH ARE ALWAYS PLACED LAST	Checked=1; otherwise=0
a) Bracelet or watch	☐
b) Intelligent clothing (e.g. pants, shirt, t-shirt, socks, hat, belt, shoe soles)	☐
c) Bathroom scale	☐
d) Toothbrush	☐
e) Fork	☐
f) Blood pressure monitor	☐
g) Pedometer	☐
h) Thermometer	☐
i) Glucose monitor	☐
j) Pill dispenser	☐
k) Pulse oximeter or spirometer	☐
l) Other connected/intelligent objects worn using a band (e.g. worn on the head, around the neck or chest, an arm, a thigh)	☐
m) Other portable connected objects (e.g. connected optical devices, connected pendants, connected hearing aids)	☐

IF Q3 = 0, GO TO Q20, OTHERWISE GO TO Q24

Section 4. Profile of the Respondent

Q20. Do you currently keep track of specific health measures or well-being information on paper in a diary or log or through manual data entry on your computer?

(e.g.: your weight, blood sugar level, blood pressure, level of physical activity, pulse, athletic performance, quality of sleep - or other aspect of your health or well-being?)

Yes	1 ☐	GO TO Q22
No	0 ☐	CONTINUE

Q21. For which of the following reasons do you not manually measure any aspect of health and well-being? **Please check all the boxes that apply to your personal situation.**

RANDOM ROTATION EXCEPT FOR ITEM "J," WHICH SHOULD ALWAYS BE LAST	Checked=1; otherwise=0
a) I am in good or excellent health, so I do not see the point.	☐
b) I would not know what information to collect or how to collect it.	☐
c) I do not have the time to be taking such measures myself.	☐
d) I am not disciplined enough for this kind of activity.	☐
e) I would not know what to do with the information collected.	☐
f) The information that my physician gives me on my health is sufficient.	☐
g) The information that my personal trainer gives me on my physical condition is sufficient.	☐
h) I have other priorities at this time in my life.	☐
i) It is of no interest to me.	☐
j) No specific reason.	☐

GO TO Q24

Q22. Generally speaking, which of the following aspects of your health and well-being do you currently keep track of or record manually on a regular basis? **Please check all the boxes that apply to your personal situation.**

RANDOM ROTATION	Checked=1; otherwise=0
a) Competition and performance in sports	☐
b) Regular physical activity	☐
c) Nutrition and eating habits	☐
d) Weight	☐
e) Quality and/or quantity of sleep	☐
f) Body temperature recording from a thermometer	☐
g) Cardiovascular, lung or respiratory airway health	☐
h) Diabetes and other metabolism-related conditions	☐
i) Medication use or treatment monitoring	☐
j) Sexual and reproductive health	☐
k) Mental and emotional health	☐
l) Oral or dental health	☐
m) Tobacco dependence	☐
n) Consumption of alcohol or drugs	☐
o) None of the above	99 ☐

CONTINUE

Q23. Generally speaking, what do you do with the data/information you manually record on your health or well-being? **Please check all the boxes that apply to your personal situation.**

RANDOM ROTATION EXCEPT FOR ITEM "F," WHICH SHOULD ALWAYS BE LAST	Checked=1; otherwise=0
a) I simply keep track of my data in my head.	▯
b) I write my data down on paper, in a personal journal or in a notebook.	▯
c) I enter and save my data in a program on my computer, tablet or smartphone (e.g. an Excel spreadsheet or a table in Word or in an app).	▯
d) I access the internet or a website to enter and save my data on a patient portal or other site (e.g. a personal patient record).	▯
e) I use some other means.	▯
f) I do not save my data.	▯

CONTINUE

Q24. How would you rate your current health status?

Excellent	Very good	Good	Rather poor	Very poor
5 ▯	4 ▯	3 ▯	2 ▯	1 ▯

CONTINUE

Q25. Do you suffer from one or more chronic conditions?

Yes	1 ▯	CONTINUE
No	0 ▯	GO TO Q27
Prefer not to answer	88 ▯	GO TO Q27

Q26. Which ones? **Please check all the illnesses that apply to your personal situation.**

RANDOM ROTATION	Checked=1; otherwise=0
a) Diabetes	☐
b) High blood pressure	☐
c) Obesity	☐
d) Heart disease (e.g. heart attack, congestive heart failure, arrhythmia, heart disease at birth, high cholesterol)	☐
e) Lung or respiratory airway disease (e.g. asthma, cystic fibrosis, chronic obstructive pulmonary diseases such as emphysema and chronic bronchitis)	☐
f) Cancer	☐
g) Bone or muscle disease (e.g. arthritis, rheumatism, osteoarthritis, osteoporosis, back pain)	☐
h) Disease of the nervous system (e.g. stroke, memory problems, Alzheimer's disease, Parkinson's disease, dementia, migraines, head trauma)	☐
i) Mental disorders (e.g. depression, bipolar disorder, anxiety, eating disorder, personality disorder)	☐
j) Chronic infectious disease (e.g. HIV/AIDS, viral hepatitis, tuberculosis)	☐
k) Addiction to tobacco, alcohol or drugs	☐
l) Prefer not to answer	88 ☐

CONTINUE

Q27. Please indicate your gender.

Woman	1 ☐
Man	2 ☐
Prefer not to answer	88 ☐

CONTINUE

Q28. What is your age group?

18 to 24 years	25 to 34 years	35 to 44 years	45 to 54 years	55 to 64 years	65 to 74 years	75 years or older
1 ☐	2 ☐	3 ☐	4 ☐	5 ☐	6 ☐	7 ☐
Prefer not to answer	88 ☐					

CONTINUE

Q29. You currently reside in which province or territory?

Alberta	1 ☐
British Columbia	2 ☐
Manitoba	3 ☐
New Brunswick	4 ☐
Newfoundland and Labrador	5 ☐
Nova Scotia	6 ☐
Nunavut	7 ☐
Northwest Territories	8 ☐
Ontario	9 ☐
Prince Edward Island	10 ☐
Quebec	11 ☐
Saskatchewan	12 ☐
Yukon	13 ☐

CONTINUE

Q30. What is your current primary occupation?

Full-time worker (35 hours/week or more)	1 ☐	**CONTINUE**
Part-time worker (less than 35 hours/week)	2 ☐	
Student	3 ☐	**GO TO Q32**
Looking for work	4 ☐	
At home full-time	5 ☐	
Retired	6 ☐	
Other	7 ☐	
Prefer not to answer	88 ☐	

Q31. Are you a health professional?

Yes	1 ☐
No	0 ☐

CONTINUE

Q32. Including yourself, how many adults and children (under 18 years of age) usually live in your primary residence?

Number of adults: ______ **(possible values: 1 to 8)**
Prefer not to answer 88 ☐

Number of children under the age of 18: ______ **(possible values: 1 to 20)**
Prefer not to answer 88 ☐
CONTINUE

Q33. What language(s) did you first learn at home when you were a child and that you still understand?

French	1 ☐
English	2 ☐
Other	3 ☐
French and English	4 ☐
French and other	5 ☐
English and other	6 ☐
Prefer not to answer	88 ☐

CONTINUE

Q34. What is your highest completed level of education?

Primary school	1 ☐
Secondary school	2 ☐
College/CÉGEP	3 ☐
Undergraduate university (certificate)	4 ☐
Undergraduate university (bachelor’s degree)	5 ☐
Graduate university (master’s degree or graduate diploma)	6 ☐
Graduate university (doctorate)	7 ☐
Prefer not to answer	88 ☐

CONTINUE

Q35. What was your total gross family income (before income taxes) in 2015?

$19,999 or less	1 ☐
$20,000 to $39,999	2 ☐
$40,000 to $59,999	3 ☐
$60,000 to $79,999	4 ☐
$80,000 to $99,999	5 ☐
$100,000 to $200,000	6 ☐
Over $200,000	7 ☐
Prefer not to answer	88 ☐

CONTINUE

Q36. What is your current postal code?

|____|____|____|____|____|____|

88 ☐ Prefer not to answer

End of the study. Thank you for your participation!
